# Solid pseudopapillary neoplasm of the pancreas: A case report with a brief literature review

**DOI:** 10.1016/j.ijscr.2024.109234

**Published:** 2024-01-10

**Authors:** Yasser ALGhabra, Mohammad Hamdi, Malath Alhomsi, Ahmad Alusef, Safaa Qatleesh, Mhd Ali Ousta

**Affiliations:** a Al-Mouwasat University Hospital, Faculty of Medicine, Damascus University, Damascus, Syria; b M.D, Faculty of Medicine, Aleppo University, Aleppo, Syria

**Keywords:** Solid pseudopapillary, Neoplasm, Pancreas

## Abstract

**Introduction:**

Solid pseudopapillary neoplasm (SPN) of the pancreas, representing only 1 % of pancreatic cancers, was identified by Virginia Frantz in 1959. Predominantly affecting young females, it often remains asymptomatic, posing diagnostic challenges due to slow growth. This paper emphasizes SPN's rarity and associated diagnostic complexities.

**Case presentation:**

In a specific case, a 17-year-old female with post-traumatic right flank pain underwent an enhanced CT scan, revealing a well-defined, hypodense mass in the pancreatic head. With normal laboratory results, a diagnostic laparotomy exposed a sizable solid cystic mass. A Whipple procedure unveiled a predominantly cystic mass enveloped by a well-developed capsule.

**Discussion:**

SPN appears as a distinct mixed solid and cystic lesion on imaging, necessitating confirmation through core biopsy. Surgical resection, the primary treatment, ensures a positive overall prognosis, despite rare recurrence and metastases. Microscopic examination reveals pseudopapillae, and immunohistochemistry aids diagnosis with positive staining for estrogen receptor, progesterone receptor, CD10, and CD99.

**Conclusion:**

SPN, a rare pancreatic neoplasm predominantly affecting young females, may present with abdominal pain or palpable mass despite its usual asymptomatic nature. Diagnosis involves imaging and biopsy confirmation, with surgical resection as the curative treatment. While prognosis is generally favorable, comprehensive understanding and improved management require further research for this uncommon pancreatic neoplasm.

## Highlights

• Solid pseudopapillary neoplasm (SPN) is a rare pancreatic neoplasm, primarily affecting young females.
• Often discovered incidentally due to slow growth, SPN may present with abdominal pain or a palpable mass.
• Accurate diagnosis is confirmed through core biopsy guided by ultrasound or CT.
• Complete surgical excision is the curative treatment with a generally positive prognosis.
• Further research is needed to enhance the understanding and management of this rare pancreatic neoplasm.

## 1 Introduction

Solid pseudopapillary neoplasm (SPN) of the pancreas, also known as “papillary cystic tumor of the pancreas,” was first described by Virginia Frantz in 1959 [1]. SPN is a rare pancreatic neoplasm, accounting for approximately 1 % of all pancreatic cancers [2]. It predominately affects females with a median age of 20–30 years [3]. According to the 2010 WHO classification, SPN is classified as a low-grade malignant neoplasm of the exocrine pancreas [4]. These tumors are often asymptomatic and are discovered incidentally during diagnostic imaging for unrelated diseases due to their slow growth [5]. When symptomatic, patients may present with non-specific symptoms such as abdominal pain or palpable abdominal mass [6]. A core biopsy with ultrasound or CT-guidance can confirm the diagnosis [7]. Total surgical resection is the primary treatment option, with an excellent overall prognosis [8]. However, recurrence and metastases may occur in a small percentage of patients. This case report has been reported in line with the SCARE Criteria [9].

## 2 Case presentation

**Fig. 1:**
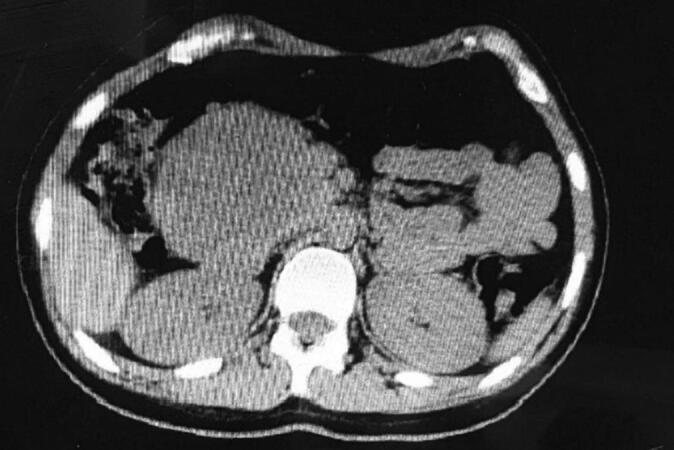
Enhanced MSCT, a hypodense well-circumscribed mass in the pancreatic head extends caudally on the anterior surface of the IVC with direct contact to the right renal artery and vein without engorgement.

A 17-year-old female with no prior medical history presented with moderate post-traumatic pain in her right flank that had persisted for two weeks. She denied experiencing any significant weight loss, loss of appetite, hemorrhagic symptoms, jaundice, nausea, vomiting, or hematuria. Physical examination revealed tenderness throughout her abdomen, but no palpable masses or organ enlargement were detected. An enhanced multislice computed tomography (MSCT) scan revealed a well-defined, hypodense mass in the pancreatic head (Fig. 1). The mass extended caudally along the anterior surface of the inferior vena cava (IVC) and made direct contact with the right renal artery and vein, but without causing any blockage or engorgement. Notably, the mass did not come into contact with the liver parenchyma. The measured size of the mass was 11 × 8 cm2, and it exhibited central necrosis. During the early phase after contrast injection, the periphery of the mass showed enhancement, while central enhancement was observed in the late phase. No lymphadenopathy, free fluid, or metastatic implants were observed. Table 1 summarizes the patient's laboratory investigations at admission. Table 1: Laboratory tests at admission.

**Table 1:** Laboratory tests at admission.

Test	Result	Reference range
White blood cell count	4.1 × 10^3^/mm^3^	4–11 × 10^3^/mm^3^
Red blood cell count	3.95 × 10^6^/μl	Female:3.5‐5.5 × 10^6^/μl
Hematocrit	32.8 %	Female: 36–46 %
RBCs MCV	83 fL	80–100 fL
Platelet count	299 × 10^3^/μl	150–400 × 10^3^/μl
BUN	10 mg/dl	7–18 mg/dl
Creatinine	0.5 mg/dl	0.6–1.2 mg/dL
Sodium	143 mmol/l	135–148 mmol/l
Potassium	4.5 mmol/l	3.5–5 mmol/l
Chloride	104 mmol/l	95–105 mmol/l
ALT (GPT)	13 U/I	Up to 31 U/I
AST (GOT)	16 U/I	Up to 32 U/I

Abbreviations: MCV = mean corpuscular volume, BUN = blood urea nitrogen.

**Fig. 2:**
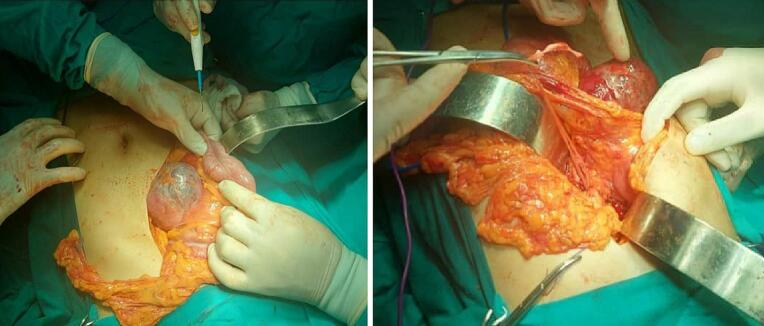
Intra-operative view of the surgical site.

**Fig. 3:**
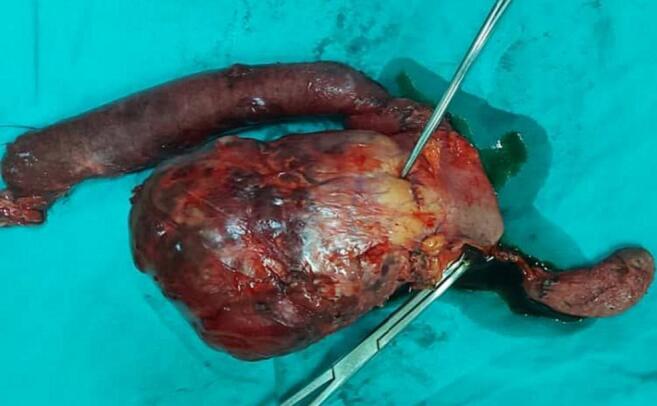
Gross examination of the Whipple resection specimen including pancreatic tumor tissue.

The patient underwent an exploratory laparotomy, during which a large solid cystic mass measuring 10 × 9 cm^2^ in diameter was discovered originating from the head of the pancreas (Fig. 2). No ascites, metastatic implants in the abdomen and pelvis, or carcinomatosis were observed. Subsequently, a Whipple procedure with pylorus-preservation was performed, and the reconstruction involved pancreatojejunostomy, hepaticojejunostomy, and gastrojejunostomy. Gross examination of the Whipple resection specimen revealed a large solitary mass in the pancreatic head, measuring approximately 7 cm2 in its largest dimension (Fig. 3). The cross-section appearance ranged from solid to predominantly cystic, with vegetations and areas of hemorrhage. The mass was surrounded by a well-developed capsule.

**Fig. 4:**
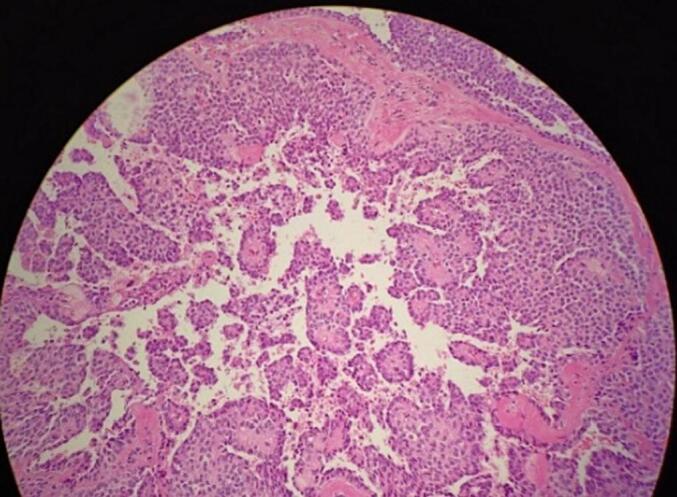
Histopathologic examination with H&E showing several layers of tumor cells lined with dedicated papillary fronds.

**Fig. 5:**
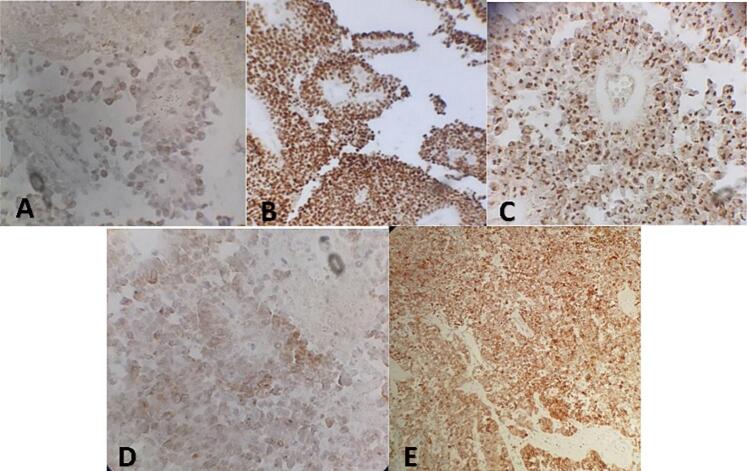
Tumor cells stained positive with ER (A), PR (B), CD 99 (C), CD10 (D), and synaptophysin (E) via IHC analysis.

The specimen was processed using standard protocols, which included fixation in 10 % buffered formalin, embedding in paraffin, and serial sectioning into slices measuring 4 μm in thickness. Hematoxylin and eosin staining were performed routinely. Microscopically, the mass exhibited both solid and cystic components. The most distinctive feature was the presence of pseudopapillae, which were structures covered by several layers of epithelial cells (Fig. 4). The cells surrounding the pseudopapillae were discohesive and exhibited fibrovascular cores. Additionally, there was evidence of cystic degeneration. Immunohistochemically, the tumor cells stained positive for estrogen receptor (Fig. 5-A), progesterone receptor (Fig. 5-B), CD10 (Fig. 5-D), and CD99 (in a dot-like pattern) (Fig. 5-C). The tumor cells also exhibited diffuse positivity for synaptophysin (Fig. 5-E), which is a marker for neuroendocrine differentiation. However, chromogranin, another neuroendocrine marker, was negative.

## 3 Discussion

Solid pseudopapillary neoplasm (SPN) of the pancreas was first reported by Virginia Frantz in 1959 as “papillary cystic tumor of the pancreas” [1]. It has also been referred to as solid and papillary epithelial neoplasms, papillary cystic tumors of the pancreas, and solid and cystic tumors. SPN is a rare neoplasm that predominantly affects young females [2,3]. It typically occurs in women under 35 years of age [2]. Incidental detection of SPN is becoming more popular with widespread use of cross-sectional imaging. The most common symptom in symptomatic adults is abdominal pain, followed by nausea, vomiting, and weight loss. Other less frequent symptoms include GI obstruction, anemia, jaundice, and pancreatitis. Patients may also have a palpable mass, which is the most common presentation in pediatrics group [10]. The finding of a mixed solid and cystic pancreatic lesion on cross-sectional imaging in a young woman is suggestive of a solid pseudopapillary neoplasm (SPN). On magnetic resonance imaging, the lesions may appear as well-demarcated solid tumors. In a study of MRI characteristics of small solid tumors of the pancreas, SPN had significantly lower signal intensity on T1-weighted images, higher signal intensity on T2-weighted images, and had early heterogeneous and progressive enhancement on MRI compared with adenocarcinomas and endocrine tumors. However, it is unclear if cystic and mixed solid and cystic SPN will have the same MRI characteristics [11]. The characteristic appearance on EUS is a well-demarcated, echo-poor, solid-appearing mass, although it can also appear as a mixed solid and cystic lesion or as a purely cystic lesion [12]. Irregular calcifications are present in up to 20 % of cases. EUS-FNA cytologic analysis reveals characteristic branching papillae with myxoid stroma, best seen in cellblock material. Cytology is diagnostic in up to 75 % of cases. Special stains, including vimentin and CD10, may be required to differentiate an SPN from a pancreatic neuroendocrine tumor (eg, insulinoma) [13,14]. The malignant potential of SPNs has not been well studied. In a series of 62 patients, nine (15 %) had malignant SPNs [15]. No factors were identified that predicted malignancy. Malignant solid pseudopapillary neoplasms (SPNs) can be cured when completely excised, and prolonged survival can be seen even in the presence of metastatic disease with surgical debulking [16,17]. Given the potential malignancy of the lesion and the curative effect of complete surgical excision, resection should be considered in most cases of a pancreatic mixed solid and cystic lesion in a young woman found on CT or MRI. Cross-sectional imaging usually reveals a large, solitary, well-circumscribed, heterogeneous lesion that can be completely cystic, mixed cystic and solid, or purely solid. The lesions are generally demarcated by a peripheral capsule, and occasional calcifications can also be observed [18]. SPNs are equally distributed throughout the pancreas. They begin as solid neoplasms that often become cystic as they grow large and the cells become so far removed their blood supply that they undergo apoptosis or necrosis. Histologically, SPN is composed primarily of polygonal cells with a moderate amount of cytoplasm. Multiple capillary-size vessels traverse the tumor, and when they become cystic, these vessels are surrounded by single or multiple layers of surviving tumor cells, giving the lining an irregular “pseudopapillary” appearance. In the older literature, these neoplasms have been called “solid-cystic tumor,” “papillary cystic neoplasm,” Hamoudi or Frantz tumors, and by many other names, reflecting their complex solid, cystic and “papillary” morphology [19,22]. Recently, SPN are classified as a low-grade malignancy [[19], [20], [21]]. Prognosis is generally good [22,23]. Among 1952 patients with SPN treated by surgical resection and with a median follow-up of 36 months, disease-free survival was 95.6 % with recurrence in 4.4 % [22]. Distant metastases were reported in approximately 8 % of cases [22]. Tumors with a high proliferation rate, as indicated by a Ki-67 index of 4 % or more, may be associated with a less favorable outcome [24]. However, the impact of a high Ki-67 index on the management of these patients remains unclear. Solid pseudopapillary neoplasms (SPNs) are typically located in the body or tail of the pancreas and often exhibit a combination of solid and cystic components, along with occasional calcifications [25,26]. Patients who undergo complete surgical resection have shown an overall 5-year survival rate of approximately 95 % [27]. The precise recurrence rate after complete tumor resection is not well established, but it may be higher in patients who undergo tumor enucleation or incomplete excision, both of which should be avoided [3]. As the mean time to recurrence was found to be approximately four years, patients treated for SPN should be monitored for at least five years [28]. Large tumors, exceeding 3.0 cm, typically exhibit rounded shapes, are well-circumscribed, and are encased by a fibrous pseudocapsule, distinctly separated from the pancreatic tissue. These larger tumors present a variegated cut surface with varying combinations of solid, hemorrhagic, and cystic-necrotic areas. Conversely, smaller tumors, less than 3.0 cm in size, often demonstrate fewer cystic changes and may lack distinct demarcation, occasionally resembling an unencapsulated structure. On the other hand, previous literature extensively describes the imaging characteristics aligning with larger solid pseudopapillary tumors; however, limited knowledge about smaller SPT remains, resulting in particular challenges in preoperative diagnosis, especially with smaller tumors [29]. Consequently, the prospect of surgical resection appears suitable for nearly all patients, irrespective of the size of the tumor mass [30]. For cases that deemed inoperable or displaying aggressive malignant characteristics, a multimodal therapeutic strategy has been contemplated. However, the application of chemotherapy and radiotherapy in the management of these low-grade tumors remains constrained due to limited empirical data. Notably, there is consideration for the resection of distant metastases both during the primary surgical intervention and in instances of disease relapse. Moreover, alternative therapeutic modalities are being explored for liver metastases, encompassing a spectrum ranging from chemotherapy, alcohol injection, chemoembolization, to radiotherapy and even liver transplantation [31].

## 4 Conclusion

Solid pseudopapillary neoplasm (SPN) of the pancreas is a rare neoplasm that mainly affects young females and is usually discovered incidentally during imaging for other conditions due to its slow growth. Symptoms such as abdominal pain or a palpable mass if present, can aid in the diagnosis, which is confirmed through a core biopsy guided by ultrasound or CT. Complete surgical excision is the curative treatment, and the prognosis is generally positive, with high rates of disease-free survival and low rates of recurrence and metastases. However, further research is needed to better understand and improve the management of this rare pancreatic neoplasm.

## Abbreviations

SPN: solid pseudopapillary neoplasm
MCV: mean corpuscular volume
BUN: blood urea nitrogen
MSCT: enhanced multislice computed tomography

## Parental consent

Written informed consent was obtained from the patient's parents for publication and any accompanying images. A copy of the written consent is available for review by the Editor-in-Chief of this journal on request.

## Ethical approval

Ethics clearance was not necessary since the University waives ethics approval for publication of case reports involving no patients' images, and the case report is not containing any personal information. The ethical approval is obligatory for research that involve human or animal experiments.

## Funding

This research cost no charge.

## Author contribution

Yasser ALGhabra: Contributed to original draft and design of the study.

Mohammad Hamdi: Contributed to original draft and publishing of the final manusctipt.

Malath Alhomsi: Contributed to collecting data of the case presentation and interpretation of the results.

Ahmad Alusef: Contributed to collecting data of the case presentation and interpretation of the results.

Safaa Qatleesh: Contributed to collecting data and editing some points.

Mhd Ali Ousta: Supervising and editing some points in the final manuscript.

All authors read and approved the final manusctipt.

## Guarantor

Ahmad Alusef.

## Declaration of competing interest

The authors have declared no potential conflicts of interest.
